# Using Cognitive Interviewing to Assess the Feasibility and Acceptability of the Network Canvas Platform for Social Network Data Collection Among Adolescent and Young Adult Survivors of Cancer: Pilot Descriptive Study

**DOI:** 10.2196/91765

**Published:** 2026-10-30

**Authors:** Pooja Rao, Ian Rowe-Nicholls, Gabrielle Lang, Tabitha Chen, Ashton Verdery, Laura Koehly, Erika VanDyke, Heather Costigan, Deepa Sekhar, Kristin Bingen, Smita Dandekar, Christina Toepke, Tonya King, Michelle Birkett, Lauren Van Scoy

**Affiliations:** 1 Department of Pediatrics, Penn State College of Medicine, 700 HMC Crescent Road, Hershey, PA, 17033, United States, 1 7175316012; 2 Department of Sociology and Criminology, Pennsylvania State University, State College, PA, United States; 3 Department of Pediatrics, National Human Genome Research Institute, National Institutes of Health, Bethesda, MD, United States; 4 Department of Medicine, Penn State College of Medicine, Hershey, PA, United States; 5 Department of Pediatrics, Medical College of Wisconsin, Milwaukee, WI, United States; 6 Kolschowsky Research & Education Institute, Sarasota Memorial Health Care System, Sarasota, FL, United States; 7 Center for Computational and Social Sciences in Health, Feinberg School of Medicine, Northwestern University, Chicago, IL, United States

**Keywords:** cognitive interviewing, social networks, social network analysis, adolescent and young adult survivors of cancer, social health

## Abstract

**Background:**

Little is known about the composition and function of the social networks of adolescent and young adult survivors of cancer, despite the importance of social health for adolescent and young adult development. For adolescent and young adult survivors of cancer, social networks may be particularly important sources of support and coping. Understanding this group’s social health through understanding their social networks can inform intervention development to improve various facets of health and life productivity.

**Objective:**

The objective of this descriptive study was to explore the feasibility and acceptability of collecting and analyzing social network data using the Network Canvas platform among a subset of adolescent and young adult survivors of childhood cancer.

**Methods:**

We used cognitive interviewing, a qualitative method to assess survey comprehension and usability, to assess the feasibility and acceptability of using the Network Canvas social network data collection platform among 10 adolescent and young adult survivors of cancer. Participants (ie, egos) were asked about people (ie, alters) in their networks before cancer diagnosis (via recall) and currently (after cancer diagnosis). Feasibility was explored in three ways: (1) the proportion of participants who completed the full Network Canvas survey; (2) qualitative analysis of cognitive interviews addressing usability and comprehension; and (3) demonstration of whether self-administered, role-anonymized responses yielded data complete enough to support the creation of standard egocentric network measures through preliminary descriptive statistics. Acceptability was explored through qualitative analysis of the desire of participants to use Network Canvas again and features they liked when using Network Canvas.

**Results:**

In terms of feasibility, all 10 participants completed the Network Canvas survey. In qualitative analysis, participants described the ease of using Network Canvas (including engagement) while also describing ways to improve its usability. In terms of acceptability, when asked if they would use Network Canvas again, participants overall answered positively.

**Conclusions:**

Network Canvas use among our sample of adolescent and young adult survivors of cancer was feasible and acceptable based on this exploratory analysis. The collection and analysis of social network data using Network Canvas was feasible in this pilot study. Additionally, the potentially therapeutic experience reported by some participants after completing the Network Canvas survey deserves further exploration. This work provides a starting point for future social health studies, psychosocial programming, and identification of those at risk of social disconnect.

## Introduction

The incidence of childhood cancer increased from 14.23 cases per 100,000 children in 1975 to 1979 to 18.89 in 2010 to 2019, with the rate of survival continuing to improve for the individuals in the latter group, who are now in the adolescent and young adult age range [1]. While psychosocial health constructs such as distress and anxiety are routinely studied [2], social health is often underaddressed in the care of this growing population of adolescent and young adult survivors of cancer, which includes adolescent and young adult survivors of childhood cancer as well as those diagnosed with cancer as adolescents and young adults. Specifically, little is known about the composition and functions of their social networks, an important component of social health. Social health is a critical part of total health per the World Health Organization [3]. Additionally, a person’s social and community context (relationships and interactions with others) is a social determinant of health [4]. Social health is associated with improved coping strategies [5,6] and mortality reduction [7]. Furthermore, the COVID-19 pandemic negatively impacted social health among youth [8], likely exacerbating social isolation and social disconnect in this already vulnerable population.

Adolescent and young adult survivors of cancer place high value on their social networks [6,9,10], yet qualitative work suggests that cancer and its treatment lead to distinct challenges for them to form and maintain relationships, especially with peers [11]. These challenges may be related to missed opportunities to socialize and form relationships due to the demands of cancer treatment. Additionally, adolescent and young adult survivors of cancer have unique developmental needs, including difficulty nurturing peer relationships outside of existing social structures such as school and extracurricular activities and navigating professional settings [12]. Understanding the social health of adolescent and young adult survivors of cancer through understanding their social networks can inform the development of impactful interventions to improve social relationships and, resultantly, health and life productivity [13]. Despite the importance of understanding this population’s social health, few studies have sought to evaluate their social networks, leaving a large knowledge gap in the social health promotion of adolescent and young adult survivors of cancer. To address this gap, social network analysis (SNA), a rigorous social science methodology, can be used to characterize the composition, connectedness, and functions of social networks [14,15]. SNA can identify network dynamics over time, such as the change in people in a network (ie, network churn, a common occurrence in adolescents and young adults’ social networks [16,17]). SNA has also been used to understand and reduce disparities in health outcomes [18]. Leveraging SNA to understand the social networks of adolescent and young adult survivors of cancer has not been routinely studied. Using SNA to understand this population’s social networks could offer a much richer, more granular, and more comprehensive assessment of various facets of their social health to better prioritize which aspects of social health to intervene on for programmatic development.

The objective of this study was to assess the feasibility and acceptability of collecting social network data using the Network Canvas platform among a subset of adolescent and young adult survivors of cancer who were survivors of childhood cancer. We chose to specifically assess the feasibility and acceptability of using this platform among this subset to understand nuances in Network Canvas survey and design and adaptations for this population. We also describe feasibility by the proportion of participants able to complete the Network Canvas survey and whether the resulting data supported the computation of standard egocentric network measures, including cross–time point comparison. Developed by the Complex Data Collective [19], Network Canvas is a National Institutes of Health–supported free and open-source software platform to collect social network data from participants through the use of interactive and visually pleasing interfaces, thereby increasing participant engagement [20]. Use of the Network Canvas platform has the potential to enhance the research and clinical care of adolescent and young adult survivors of cancer.

## Methods

### Network Canvas Background

Participants completed a social network survey using Network Canvas [21]. This platform has been rigorously tested to ensure usability on both the deliverer and recipient interfaces [22]. It was designed for researchers new to social network assessment and analysis to be able to create tailored network surveys for their study participants. As an alternative to other cumbersome methods, Network Canvas allows participants to interact with their own social network while inputting their data into the program, prompting participant engagement and increasing efficiency for the research team [20]. We specifically chose to use Network Canvas to collect our social network data to increase participant engagement, minimize study team labor, and determine future clinical and research applications for Network Canvas data collection.

In the survey, we asked a series of name generators, soliciting responses about who the participant (ie, ego) was close to, with whom they talked about various health topics, and weak ties to generate a list of people in their social network (ie, alters). Extensive literature establishes the validity of this social network data collection approach, including among adolescents [23]. We also recognized the need to reduce any potential risks that might arise from unauthorized disclosure of personal data about alters (secondary participants) [24]. To preserve the anonymity of collected alter data, we asked participants to name the alter role (eg, mother) or initials for friends (eg, PR) rather than specific full alter names.

### Design and Procedure

To explore the feasibility and acceptability of Network Canvas among adolescent and young adult survivors of cancer, our team conducted 10 participant interviews. Each interview consisted of completing the Network Canvas survey along with a semistructured cognitive interview and postsurvey interview. These data were qualitatively analyzed to assess feasibility and acceptability. We also describe feasibility quantitatively by the proportion of participants able to complete the Network Canvas survey. Additionally, we describe data collection and analysis feasibility through a preliminary analysis of broad social network descriptives.

Study visits were conducted in person and recorded using Microsoft Teams, which captured both the interview audio and on-screen activity during completion of the Network Canvas survey. The survey consisted of several steps or stages. The first set of questions assessed participant sociodemographic information. Participants were then asked to provide information about alters in their social networks both before their diagnosis (via recall) and after they were diagnosed with cancer (ie, current network members). To populate their networks, participants listed alters they were “close to” both before their cancer diagnosis and currently, as well as alters with whom they talked about various aspects of their health (physical, emotional and mental, and religious and spiritual) both before their cancer diagnosis and currently. Participants were also asked about weak ties (ie, those with whom they spoke about important topics but had not named before) in their networks. For each named alter role, participants indicated the types of social support (informational, financial, and emotional) they provided to them both before their cancer diagnosis and currently. After eliciting alters by their roles, participants were asked about sociodemographic information for each alter, as well as activities they did and topics they talked about with named alters. Participants were asked about modes and frequencies of communication with alters. Participants then arranged alters in a visual social network diagram (ie, sociogram) and defined the existence and intensity of connections between pairs of alters (ie, whether alters knew each other and were close to each other). Finally, participants selected alters with whom relationships became stronger or weaker currently (ie, after they were diagnosed with cancer).

To comprehensively understand the experience of adolescent and young adult survivors of cancer using the Network Canvas platform, we used cognitive interviewing, a qualitative method, to assess Network Canvas usability (including feasibility and acceptability). Cognitive interviewing involved 2 components: think-aloud methodology and verbal probing [25]. Using think-aloud methodology, participants verbalized their thoughts in real time as they navigated through the survey, noting what was clear or confusing, identifying which prompts needed clarification, and reporting any technical issues encountered. Think-aloud interviewing has been previously used for evaluating the feasibility and acceptability of Network Canvas use in other study populations [26]. We then conducted individualized semistructured interviews directly after completion of the Network Canvas survey. These interviews assessed participants’ comprehension of survey questions and identified features they would change (if any). Additional details about qualitative data rigor are provided in Multimedia Appendix 1.

Feasibility was explored in three ways: (1) the proportion of participants who completed the full Network Canvas survey; (2) qualitative analysis of cognitive interviews addressing usability and comprehension; and (3) demonstration of whether self-administered, role-anonymized responses yielded data complete enough to support the creation of standard egocentric network measures through preliminary descriptive statistics. Acceptability was explored through qualitative analysis of the desire of participants to use Network Canvas again and features they liked when using it.

### Ethical Considerations

This study was approved by the Pennsylvania State University Institutional Review Board (STUDY00026588). Written informed consent was obtained from all participants prior to their participation, and all participants voluntarily agreed to take part in the study. As outlined above, secondary participants (ie, those who were named by consented study participants) were included with waived consent per federal regulations (Title 45 of the Code of Federal Regulations part 46.116(f)) following typical guidelines for ethical considerations in the social network and health literature [24,27,28]. To enhance confidentiality and minimize risk, all data were securely stored in password-protected and approved databases to protect personal information and ensure confidentiality. Participants received a compensation of US $40 for taking part in the study.

### Recruitment

Participants were eligible if they were aged 18 to 29 years at the time of the interview, had a history of cancer, were receiving survivorship care at our institution, and were fluent in written and spoken English. We focused on the age range of 18 to 29 years, an age group known as emerging adults [12], as they are distinct developmentally from both younger patients under 18 years with cancer [29] and older patients aged 30 to 39 years within the National Cancer Institute designated age range for adolescent and young adult survivors of cancer, with developmentally unique neurocognitive, relational, and emotional challenges [30-34]. Exclusion criteria included participants who were non–English speaking and those with cognitive inability to take part in the study as determined by the study team.

Participants were identified through review of the electronic medical record and clinic templates from our institutional Childhood Cancer Survivorship Program for child, adolescent, and young adult survivors of cancer. Participants were approached for consent in person. The study team verbally reviewed the consent form with the participants, and participants signed it if they agreed to take part. Data collection took place immediately after consenting. Data collection was stopped after 10 participants were enrolled.

### Analysis

Cognitive interview data were analyzed using summative thematic development through a 3-tiered review process: initial summary, verification by the primary interviewer, and independent review by other members of the research team with qualitative experience. The team systematically reviewed participant responses to identify patterns in feasibility (including usability and comprehension) and acceptability. Themes were organized into categories addressing technical functionality, content clarity, and overall user experience, with representative quotes supporting each theme. Multimedia Appendix 1 provides the COREQ (Consolidated Criteria for Reporting Qualitative Research) reporting guidelines on qualitative rigor, and Multimedia Appendix 2 provides the interview guide used for cognitive interviewing.

Social network descriptives were analyzed in R (R Foundation for Statistical Computing) using *tidyverse*-based scripts [35] for data cleaning and summary statistics. We processed Network Canvas survey exports (participants’ lists of network members) to construct various measures characterizing participants’ social networks (both before cancer diagnosis and currently). These include network size and change over time (before cancer diagnosis to currently), relationship closeness and duration, and network composition (relationship type and demographics).

## Results

### Demographics

Participant demographics are shown in Table 1. Of the 10 participants, 6 were female, 9 identified as White individuals, and none were Hispanic or Latino individuals. Eight of the 10 participants had hematologic malignancies, with the remaining 2 participants having a history of hepatoblastoma. All had completed cancer treatment. The median time since diagnosis was 16.4 (IQR 14.1-17.9) years. Of note, 1 participant (participant 9) was approached for enrollment but ultimately did not enroll given that we were able to recruit and enroll the remaining participants before participant 9 completed data collection.

Qualitative themes were organized related to feasibility and acceptability of using the Network Canvas platform. Quotes were edited minimally for clarity and brevity.

**Table 1.** Demographic characteristics (N=10).

	Values
Age at interview (y), median (IQR; range)	20.8 (19.9-21.6; 18.7-29.3)
Age at diagnosis (y), median (IQR; range)	4.9 (2.9-6.5; 1.3-17.7)
Time since diagnosis (y), median (IQR; range)	16.4 (14.1-17.9; 10.3-19.6)
Participant identified sex, n (%)	
Female	6 (60)
Male	4 (40)
Race (participants could check all that applied), n (%)	
Asian or Pacific Islander	1 (10)
Black or African American	2 (20)
White or Caucasian	9 (90)
Ethnicity, n (%)	
Not Hispanic or Latino	10 (100)
Cancer diagnosis, n (%)	
Acute lymphoblastic leukemia	5 (50)
Acute promyelocytic leukemia	1 (10)
Hepatoblastoma	2 (20)
Hodgkin lymphoma	2 (20)
Treatment status, n (%)	
Completed treatment	10 (100)
Rurality^a^, n (%)	
Rural	4 (40)
Nonrural	6 (60)

a Rurality was determined through the Centers for Medicare and Medicaid Services rural health clinic program.

### Feasibility

All 10 enrolled participants were able to complete the Network Canvas survey. Overall, participants found the platform engaging and interactive. One participant said the following:

I’m liking it. It’s just fun to look at. It really is, and it’s easy to get around.

[Interview 7]

They further elaborated the following:

...I love how interactive it is. I really do. It makes it fun. Like, it really makes it fun. Especially, like you said, since it’s a long survey...this little interaction part right here... [Explicit]. It’s a lot of fun.

[Interview 7]

Another participant agreed:

Everything was pretty coherent.... I like the display. I like the color, it’s very nice, to be quite honest. It flows very easily.

[Interview 11]

Participants were given the option to use the touch screen or a screen pen to complete the survey. Participants had mixed feelings about the usability of the screen pen based on different survey sections:

For [a] signature, it’s definitely easier to have a pen. But if it’s just typing your name, a keyboard is fine.

[Interview 1]

Participants also discussed that certain features, such as the “drag and drop” capabilities for organizing responses into buckets (rather than using lists to check off options), were engaging. For example, in response to completing a screen where participants organized alters into relationship types, one participant said the following:

I like the dragging around. I think it engages more than just a drop down list one. And I think I keep saying that. I just like the active participation....

[Interview 4]

Several participants appreciated having a research team member present to help them with clarifications and explanations with some navigation components:

So, I just think it needs some just clear instruction, stuff like that. And I don’t know if there’s a way to change the names of these, but the names of them might be a little confusing just because they pop up and you’re like, what am I looking at? And then instructions because I’m like, what do I do next? And then I’m like, I don’t know what I’m doing. So, what do I do?

[Interview 2]

To improve usability, participants suggested simplifying survey language and providing clearer time point distinctions. For example, participants whose cancer diagnosis had been several years before found it difficult to differentiate between “before cancer diagnosis” and “current” networks given the longer recall times. Additionally, participants recommended using sensitive language when asking about network members who passed away between the pre–cancer diagnosis and current network assessments.

### Acceptability

When asked whether they would use the Network Canvas platform again in the future for another study, participants overall answered positively. One participant said the following:

It’s very engaging, to be honest, because usually, typically, surveys that just give you a pen and paper, which can get very boring, especially when you have to fill out a bunch of stuff. I think having to do the stuff that’s a little bit more interesting, especially with more colors and then having to drag and drop stuff, doing stuff a little bit more interactive, makes things more engaging. Especially when you [are] filling out stuff that’s a little bit tedious.

[Interview 11]

Another agreed:

What I liked most about the survey is that it was unique. It wasn't boring.

[Interview 10]

Participants also described the potential therapeutic experience of being able to complete the survey because it gave them an opportunity to reflect on their social networks. One participant described how completion of the study could supplement clinical aspects of medical care to help their social health:

I think it would help them realize their networks on a more—being able to see it in front of them. I think if I was given this tool when I was sick 10 years ago, I think it could have helped me identify my feelings better. And it also might be a useful tool if someone’s in therapy, you can physically show your therapist what’s going on in your head as to your relationships with people. So it might be useful from that aspect as well.

[Interview 4]

Another agreed and described how completion of the survey served as a way to self-reflect on their social experiences related to their personal cancer diagnosis:

It really makes you reflect.... So...they can only think of three people off the top of their head. ...That could show them, like, I'm either shutting too many people out, I'm not trying to get out there, or that I really just need to get out there more. I think that’s what this could do. Like, it could be an eye-opener for some people.... Because I did.... Being a cancer patient, I used to...shut people out for a while.... So, seeing this and thinking, like, dang. Like, yeah, like, I did [shut] a lot of people out. Like, there’s a lot more people that I could have put down, that I could have thought of. But no, so I definitely think that it’s a lot of self-reflecting with that.

[Interview 7]

### Preliminary Social Network Descriptives

As a measure of data collection and analysis feasibility, we report preliminary social network descriptives.

Participants were diagnosed at a median age of 4.9 (IQR 2.9‐6.5) years, with all participants being diagnosed before the age of 18 years. At the time of the interview, participants had a median age of 20.8 (IQR 19.9‐21.6) years, representing a median of 16.4 (IQR 14.1‐17.9) years since their initial diagnosis (Table 1). Across the study, participants reported a median of 6.5 (IQR 4.25-8.785) alters per network (Table 2). Relationship composition also shifted, with networks becoming more centered on friends and partners over time. The median number of reported friends and partners increased from 0.5 (IQR 0‐1) before diagnosis to 2 (IQR 1‐3) currently (Table 2).

**Table 2.** Network size, closeness, and duration of relationships before cancer diagnosis and currently.^a^

	Alters, median (IQR)	Close alters (%)	Alters known for >5 years (%)
All alters			
Before cancer diagnosis	4 (3.25‐5)	95.9	100
Currently	6 (4.25‐7.75)	81.4	89.8
Total	6.5 (4.25‐8.75)	87.5	91.7
Friends and partners only			
Before cancer diagnosis	0.5 (0‐1)	100	100
Currently	2 (1-3)	66.7	76.2
Total	2 (1‐3.75)	75	79.2

a Median numbers of alters are participant-level summaries reported as median (IQR) across the 10 participants. Percentages are pooled alter-level proportions, calculated by dividing the number of alters meeting the specified criterion by the total number of alters in the corresponding row. For the precancer diagnosis and current rows, the denominator includes all alters reported at that respective timepoint. The Total row represents unique alters reported at either timepoint; alters reported at both timepoints are counted once. For the Total row, an alter was classified as close if identified as close at either timepoint. “Friends and partners only” is restricted to alters classified as friends or partners.

## Discussion

### Principal Findings

The social health of adolescent and young adult survivors of cancer is an area of interest within the cancer community given its associations with various facets of health. A personal cancer diagnosis often afflicts adolescents and young adults at critical junctures of age-appropriate social development, and being able to understand these changes and areas of unmet need in their social health could have significant implications for this population’s well-being. To our knowledge, this is one of the first studies to assess the feasibility and acceptability of using the Network Canvas platform among adolescent and young adult survivors of cancer. Similarly to other studies assessing the feasibility and acceptability of the Network Canvas platform [26], our adolescent and young adult participants found Network Canvas to be user-friendly and engaging. Allowing participants to input their own data streamlined the data collection process for the research team and proved equally advantageous for the users.

One particularly interesting finding in our study was the potential therapeutic experience that some participants reported after completing the Network Canvas survey. Participants described the process of recalling and visualizing their social networks as reflective, eliciting positive emotions, and possibly offering a means of psychosocial intervention. Other similar processes have been found to be potentially therapeutic, such as participating in qualitative research studies [36]. The potential therapeutic experience of being able to reflect on one’s social relationships and gain insights from survey engagement deserves further exploration in future studies.

Additionally, for a technologically savvy adolescent and young adult population, using Network Canvas as a data collection tool holds promise for future social network studies. Participants described the clinical applications of completing a Network Canvas survey. They noted how these data could support clinical instruments to assess social disconnect among adolescent and young adult survivors of cancer, complement other sources of psychosocial support, and allow for longitudinal tracking of social network composition and function. These applications would enable adolescent and young adult survivors of cancer who are at risk of social disconnect to be referred to supportive services in a proactive, normative manner. Use of Network Canvas as a tool in clinical settings is an area of further investigation for our team.

Our preliminary social network data descriptives are also a first for adolescent and young adult survivors of cancer. Preliminarily, we found differences in our participants’ reported current social networks compared to those without a cancer history in terms of number of peers. Peer relationships are critical in adolescence and young adulthood as literature suggests that adolescent peer friendship quality is an important predictor of adult peer and romantic outcomes as well as psychosocial health, even more so than parental relationships [37]. The median number of friends and partners in the current networks of adolescent and young adult survivors of cancer (2, IQR 1‐3) was less than the average of 4 to 6 friends or best friends reported in the literature for young people as a whole; however, existing data have looked at the average number of friends among young people younger than 18 years [38,39]. This decrease in *quantitative* number of peers, coupled with our prior *qualitative* work finding negative changes in peer relationships after a personal cancer diagnosis [11], reveals that more research is needed for understanding the peer networks of adolescent and young adult survivors of cancer and justifies the need to focus on ways to promote meaningful peer relationships (not necessarily more peer relationships) to improve their health and quality of life. Nonetheless, the small, nonrepresentative sample; retrospective prediagnosis reports; and absence of a comparison group mean that apparent differences should be interpreted cautiously. SNA-based assessments using Network Canvas can further investigate these peer-based network changes in the social networks of adolescent and young adult survivors of cancer.

### Limitations

While this study had several strengths, we acknowledge several limitations. Prediagnosis data relied on participants’ retrospective recall (often at a time when participants were quite young), which grows less reliable as time since diagnosis increases. Because prediagnosis networks were reconstructed retrospectively and no comparison group was included, apparent differences may reflect recall error, normative development, or other factors and cannot necessarily be attributed to cancer or its treatment. Our social support measures also emphasized informational, financial, and emotional support and did not capture ambivalent, conflictual, or constraining relationship dynamics. The small convenience-based sample size limited our ability to detect statistical significance, and therefore, this study is an exploratory and descriptive analysis of feasibility and acceptability. Recruitment through our institutional Childhood Cancer Survivorship Program limits the generalizability of the study results. Recruiting at our survivorship program also creates potential for selection bias as participants were adolescent and young adult survivors of cancer engaged with health care and comfortable discussing the sensitive topics of social health and challenges and may not reflect the broader experience of those adolescent and young adult survivors of cancer who are more socially disconnected. This same selection may also explain participants’ positive evaluations of the Network Canvas survey as those less comfortable discussing social relationships were likely underrepresented, potentially inflating acceptability ratings relative to the broader population of adolescent and young adult survivors of cancer. Finally, navigation issues raised by adolescent and young adult survivors of cancer could be reflective of our study team’s design of the survey and not related to features of the Network Canvas platform itself.

### Conclusions

In conclusion, Network Canvas was found to be a feasible and acceptable platform to gather social network data among adolescent and young adult survivors of cancer in this exploratory analysis. The platform has implications for future research and clinical care in this population, including leveraging participants’ experiences of finding a potential therapeutic experience in engaging with the platform to reflect on their own social experiences and changes following a personal cancer diagnosis. The platform may also hold potential for use in clinical settings to gauge longitudinal social connect and disconnect, as well as in psychosocial intervention development with the aim of improved social health. This work provides a starting point for future studies, psychosocial programming, and identification of those at risk of social disconnect.

## Supplementary material

10.2196/91765 Multimedia Appendix 1 COREQ reporting guidelines.

10.2196/91765 Multimedia Appendix 2 Interview guide.

## Abbreviations

COREQ: Consolidated Criteria for Reporting Qualitative Research
SNA: social network analysis
