# Supplementary material for: Using Cognitive Interviewing to Assess the Feasibility and Acceptability of the Network Canvas Platform for Social Network Data Collection Among Adolescent and Young Adult Survivors of Cancer: Pilot Descriptive Study
Source: JMIR Form Res. 2026 Oct 30;10:e91765. doi: 10.2196/91765 (PMC13722095; doi:10.2196/91765)
Supplement: Supplementary file 1 [file formative-v10-e91765-s001.docx]

**Appendix 1: COREQ Reporting Guidelines**

**Domain 1: Research Team and Reflexivity**

*Personal characteristics:* Staff 1 (KF) is new to qualitative and mixed methods research. She, along with Staff 2 (EVD) analyzed the cognitive interview data using summative theme development.

Staff 2 (EVD) has multiple years of experience in qualitative and mixed-methods research and project management. Given her ample experience in conducting interviews and focus groups, she realizes the importance of objectivity during design and analysis because of possible bias with connections to the topic. She has prior experience working with the adolescent/young adult cancer survivor (AYACS) patient population and understands the sensitivity of the topics discussed in these interviews. She led the initial summative theming of cognitive interview data together with Staff 1.

Staff 3 (HC) also has multiple years of experience in qualitative and mixed-methods research and project management. She supervised Staff 7 in conducting the cognitive interviewing. She also reviewed and refined the summative theming analyzed by Staff 1 and 2.

The lead qualitative researcher (Staff 4- LVS) is an established physician scientist whose research interest focuses on end of life and communication issues. Her qualitative work generally follows a pragmatic approach using descriptive methods to understand phenomena, and she applies a humanistic perspective to data analysis. She oversaw cognitive interview data analysis.

Staff 5 (PR), a clinician-scientist, is the principal investigator of this project. Her research interests, largely informed by the clinical care she provides to AYACS, focuses on better understanding of the psychosocial issues of AYACS to devise effective interventions to improve the psychosocial health of this patient population. Keenly aware of her potential for bias, she purposely did not participate in interview conduct for this study, nor the initial summarization of cognitive interview data. She participated in refinement after initial summative theme development by Staff 1, 2 and 3. All staff concurred on the final themes for this manuscript.

Staff 6 (GL) is a clinical research study coordinator. She screened participants for this study.

Staff 7 (TC) is a clinical research study coordinator primarily responsible for regulatory compliance. Additionally she recruited participants for the Network Canvas study. Staff 7 also worked with Staff 3 in conducting the cognitive interviews.

(i) *Relationship with participants:* As Staff 5 either provides clinical care for or knows many of the AYACS in this study, she proactively did not conduct interviews or summative theming, to maintain data objectivity.

**Domain 2: Study Design**

(i) *Theoretical framework:* To comprehensively understand the experience of AYACS using the Network Canvas platform, we used cognitive interviewing, a qualitative method to assess Network Canvas usability (including feasibility and acceptability). Cognitive interviewing involved two components: think-aloud methodology and verbal probing. Using think-aloud methodology, participants verbalized their thoughts in real-time as they navigated through the survey, noting what was clear or confusing, identifying which prompts needed clarification, and reporting any technical issues encountered. Think-aloud interviewing has been previously used for evaluating feasibility and acceptability of Network Canvas usage in other study populations. We then conducted individualized semi-structured interviews directly after completion of the Network Canvas survey. These interviews assessed participants’ comprehension of survey questions, and identified features they would change (if any).
(ii) *Participant selection:* Participants were eligible if they were 18-29 years old at time of interview, had a history of cancer, were receiving survivorship care at our institution, and were fluent in written and spoken English. We focused on the age range of 18-29 years old, an age group known as emerging adults as they are distinct developmentally from both younger patients <18 with cancer, and also from older patients ages 30-39 within the NCI designated adolescent/young adult cancer survivor age range, with developmentally unique neurocognitive, relational and emotional challenges. Exclusion criteria included participants who were non-English speaking, and those with cognitive inability to participate in the study as determined by the study team.
(iii) *Setting:* Participant approach, consent and data collection was conducted at our institutional Childhood Cancer Survivorship Program for survivors of childhood, adolescent and young adult cancers. Study visits were conducted in person and recorded using Microsoft Teams, which captured both the interview audio and on-screen activity during completion of the Network Canvas survey.
(iv) *Data collection:* Participants were identified through review of the electronic medical record (EMR) and clinic templates from our institutional Childhood Cancer Survivorship Program for survivors of childhood, adolescent and young adult cancers. Participants were approached for consent in-person. The study team verbally reviewed the consent with the participant, and participants signed the consent if they agreed to participate. Data collection occurred immediately after consenting. Data collection was stopped after 10 participants were enrolled.

**Domain 3: Analysis and Findings**

(i) *Data analysis:* Cognitive interview data were analyzed using summative thematic development by a three-tiered review process: initial summary, verification by the primary interviewer, and independent review by other members of the research team with qualitative experience. The team systematically reviewed participant responses to identify patterns in feasibility (including usability, comprehension), and acceptability. Themes were organized into categories addressing technical functionality, content clarity, and overall user experience, with representative quotes supporting each theme.
(ii) *Reporting:* The manuscript discusses the themes that arose from coding and theming. Supporting participant quotes are provided. Quotes were minimally edited for clarity when appropriate.
