# Supplementary material for: Using Cognitive Interviewing to Assess the Feasibility and Acceptability of the Network Canvas Platform for Social Network Data Collection Among Adolescent and Young Adult Survivors of Cancer: Pilot Descriptive Study
Source: JMIR Form Res. 2026 Oct 30;10:e91765. doi: 10.2196/91765 (PMC13722095; doi:10.2196/91765)
Supplement: Supplementary file 2 [file formative-v10-e91765-s002.docx]

**Appendix 2: Interview Guide**

(prompt cited from <https://www.sciencedirect.com/topics/computer-science/think-aloud-protocol>)

PROMPT: As you participate today, I would like you to do what we call “think out loud.” What that means is that I want you to say out loud what you are thinking as you work on this survey which uses an application called Network Canvas. Let me show you what I mean. I am going to think out loud as I try to send a text message on this phone: "OK, I am looking at the home screen of the phone. I would expect there to be an icon that says “text” or “messages” right here on the home screen, but I don’t see that here. I’m confused by that. I’m going to look in other places for it. I think I’ll try to click on this icon of a callout bubble because that seems like it might be related to talking, like “I talk, the other person talks.” Okay, now that that is open, I see an icon that says “text messages.” I’m glad to see that there because that is what I was expecting to see."

Do you see what I mean about thinking out loud? Now let’s have you practice by telling me out loud how you would send an e-mail on this phone.

Great! Let us get started on filling out this Network Canvas survey. We will audio record you as you think out loud what you are thinking as you work on this survey. Are you okay with being audio-recorded today?

START AUDIO RECORDING

State date, time, Participant ID number, and Interviewer initials.

Okay the recording has started. Could you confirm for the recording that you are consent and are okay with being audio-recorded today? Great, now I’m going to ask you to go through the survey. We will be pausing after each slide to get your feedback and I encourage you to “Think out loud” as you are going through.

- Pause after each slide, encourage participant to ‘think out loud’ and explain what they are doing as they complete each slide
- Was there anything unclear on this slide/question? How would you make it clearer?
- What worked on this slide?
- What did you like about this slide?
- What did you not like about this slide?

*Once completed Network Canvas survey:*

PROMPT: Thank you for completing this survey! We have some questions we would like to ask you.

- How was your experience using the Network Canvas application for this study?
- Did you find using Network Canvas to be easy or hard to use? Tell us more.
- Anything you would do to make using Network Canvas easier? Tell us more.
- Would you be interested in using Network Canvas again in the future for another study? Tell us more.
- How would you describe using the Network Canvas application to someone else? Tell us more.
- Would you recommend using the Network Canvas application to someone else? Tell us more.
- Now I would like you to imagine if you had to complete this Network Canvas survey on your own at home through a URL link that we send to your email. Would you find that challenging, or easy? Tell us more.
- We are interested in making sure that our Network Canvas surveys can be used by adolescent and young adult cancer survivors in rural and non-rural settings.
  - Is there anything you can think of that may make it easier or harder for someone in a rural setting to complete this survey? For example, would internet services (e.g., broadband) be something to consider for delivering a survey like this that you would complete on your own at home through a URL link?
  - Are there questions on the survey that you would revise to be better suited for participants who live in rural settings versus those who do not? Tell us more.
- Is there anything else you can think of that might be a challenge for using Network Canvas in adolescent and young adult cancer survivors that we did not already touch on?

STOP AUDIO RECORDING
